# Ontogeny of biochemical, morphological and functional parameters of synaptogenesis in primary cultures of rat hippocampal and cortical neurons

**DOI:** 10.1186/s13041-015-0099-9

**Published:** 2015-02-15

**Authors:** Joshua A Harrill, Hao Chen, Karin M Streifel, Dongren Yang, William R Mundy, Pamela J Lein

**Affiliations:** US EPA Integrated Systems Toxicology Division, National Health and Environmental Effects Research Laboratory, Research Triangle Park, NC USA; Department of Molecular Biosciences, University of California-Davis, School of Veterinary Medicine, Davis, CA USA; Current address: Center for Toxicology and Environmental Health (CTEH), 1620 North Shore Drive, North Little Rock, AR 72118 USA

**Keywords:** Cortical neurons, Excitatory synapses, Inhibitory synapses, Hippocampal neurons, Primary neuronal cell cultures, Synaptogenesis

## Abstract

**Background:**

Synaptogenesis is a critical neurodevelopmental process whereby pre- and postsynaptic neurons form apposed sites of contact specialized for chemical neurotransmission. Many neurodevelopmental disorders are thought to reflect altered patterns of synaptic connectivity, including imbalances between excitatory and inhibitory synapses. Developing rapid throughput approaches for assessing synaptogenesis will facilitate toxicologic and drug screening studies of neurodevelopmental disorders. The current study describes the use of high-content imaging to quantify the ontogeny of excitatory and inhibitory synapses using *in vitro* models of neurodevelopment. These data are compared to biochemical and functional measures of synaptogenesis.

**Results:**

The ontogenetic patterns of synapse formation were compared between primary rodent hippocampal and cortical neurons over 28 days *in vitro* (DIV). As determined by ELISA, the increase in synaptophysin expression levels as cultures matured was similar between hippocampal and cortical cultures. High-content imaging of immunoreactivity of excitatory and inhibitory synaptic biomarkers demonstrated an overall greater number of synapses in hippocampal relative to cortical neurons with marked differences in the pattern of inhibitory synapse development between these two neuronal cell types. Functional assays revealed that both the mean firing rates and mean bursting rates were significantly increased in cortical cultures relative to hippocampal cultures. This difference may reflect decreased inhibitory synaptic tone in cortical *versus* hippocampal cultures.

**Conclusions:**

These data demonstrate differences and similarities in the ontogeny of synaptogenesis between hippocampal and cortical neurons, depending on the biological level examined. Assessment of synaptophysin protein levels by ELISA showed a general increase in synapse formation in both cell types with increasing time in culture, while high-content imaging was able to delineate cell type-dependent differences in formation of excitatory *versus* inhibitory synapses. The functional significance of differences in the balance of excitatory to inhibitory synapses was confirmed by the assessment of network activity using microelectrode arrays. These results suggest that high-content imaging and microelectrode arrays provide complementary approaches for quantitative assessment of synaptogenesis, which should provide a robust readout of toxicologic and pharmacologic effects on this critical neurodevelopmental event.

## Background

Synaptogenesis is a developmental process in which neurons form specialized sites of contact that mediate intercellular communication via release of pre-synaptic neurotransmitters that bind to and modulate the activity of postsynaptic neurotransmitter receptors. Synapses can be either excitatory (synaptic neurotransmission results in an excitatory postsynaptic potential) or inhibitory (synaptic neurotransmission triggers an inhibitory postsynaptic potential). The balance between excitatory and inhibitory neurotransmission is critical for proper development and function of the central nervous system [1]. Abnormalities in inhibitory synaptic function have long been implicated in the pathogenesis of epilepsy and other seizure disorders [2], and recent hypotheses of the pathogenesis of at least some neurodevelopmental disorders (i.e., autism spectrum disorders) implicate imbalance of inhibitory and excitatory neurotransmission as a causal factor [3]. Thus, developing high-throughput approaches for quantifying excitatory *versus* inhibitory synaptogenesis is becoming increasingly important for mechanistic, toxicologic and drug screening studies of neurodevelopmental disorders.

Excitatory and inhibitory synapses are distinguished by the type of neurotransmitter that is released from the presynaptic terminal and by the profile of pre- and postsynaptic proteins within the synapse. For example, in the mature central nervous system (CNS), glutamatergic synapses are excitatory and are characterized by the release of glutamate from the presynaptic terminal, the presence of vesicular glutamate transporter 1 (vGLUT1) in the presynaptic vesicle pool and the presence of postsynaptic density 95 (PSD95) in the postsynaptic density [4,5]. In contrast, mature GABAergic synapses are inhibitory and are characterized by the release of γ-aminobutyric acid (GABA) from the presynaptic terminal, the presence of vesicular GABA transporter (vGAT) in the presynaptic vesicle pool and the presence of gephyrin in the postsynaptic density [6]. Excitatory and inhibitory synapses have distinctly different roles in controlling nervous system function and may be differentially susceptible to events that modulate nervous system development, such as chemical exposure or pharmacologic intervention. Therefore, independent measurements of these two types of synapses are important for understanding how perturbations in neurodevelopmental processes affect the formation of a mature synaptic network.

A number of methods have been used to measure synaptogenesis *in vitro*, including quantification of synaptic protein levels using antibody-based methods (e.g., ELISA, western blotting and immunocytochemistry) and functional assessment using electrophysiology [7-9]. However, the relationship between these measurements of synaptogenesis at different biological levels is unclear. For example, does an increase in expression of excitatory presynaptic proteins necessarily correlate with an increase in excitatory synaptic function? In addition, the majority of published studies of synaptogenesis have relied on low-throughput approaches that assess a small number of single cells (e.g., imaging of immunostained cells or electrode recordings) or single cultures (e.g., ELISA or western blotting). Recently, high-content imaging (HCI) has been used to rapidly quantify the development of synapses *in vitro* at the cellular level based on immunocytochemical localization of synaptic proteins and to detect chemical-induced neurotoxicity [10,11]. Significant advantages of HCI include not only the increase in throughput relative to more conventional approaches, but also that it provides automated standardized acquisition of a very large number of images, which increases statistical power and removes the selection bias inherent with the more conventional single cell methods of assessing synaptogenesis. At the functional level, microelectrode arrays (MEAs) have been developed to rapidly assess the development of neuronal activity and network formation *in vitro* [12].

In the present study, we examined the ontogeny of synaptogenesis in two widely used *in vitro* models of neurodevelopment: primary cultures of rat cortical and hippocampal neurons. Synapse formation was measured over 28 DIV at differing levels of biological complexity: 1) at the molecular level using ELISA to quantify the level of synaptophysin protein; 2) at the cellular level using HCI to quantify the immunoreactivity of excitatory and inhibitory synaptic biomarkers; and 3) at the functional level using MEA recordings. Our data demonstrate quantitative and qualitative similarities and differences in measures of synaptogenesis across methods and cell types.

## Results

### Cell densities

Ideally, the same plating density of cortical and hippocampal neurons would be used across all the biological levels of synaptogenesis examined; however, results of pilot experiments (data not shown) indicated that this strategy was not feasible. Rather, plating densities needed to be optimized for each cell type in each platform. For example, the basal rate of neuronal cell loss in 96-well cultures was significantly greater in cortical cultures relative to hippocampal cultures. Cortical cultures plated at the same density as hippocampal cultures (31,250 cells/cm^2^) did not contain a sufficient number of viable neurons to examine excitatory and inhibitory synaptogenesis by HCI at time points past 7 DIV. Therefore, the plating density for cortical neurons was increased for both ELISA and HCI experiments. Likewise, cortical and hippocampal cultures seeded on MEAs at densities comparable to those used in HCI experiments did not develop sufficient levels of synaptic activity to enable reliable quantification. Thus, we adopted a plating method in which cells are restricted to a drop of medium centered on the MEA, which had previously proven to reliably support the development of synaptic network activity [13]. This method results in seeding densities approximately 5 times higher than those used in ELISA or HCI experiments. However, all other culture variables (pups from which cultures were derived, cell dissociation procedures, culture medium, substrate) were the same for both hippocampal and cortical cultures across all platforms used to quantify synaptogenesis.

### Synaptophysin ELISA

**Figure 1:**
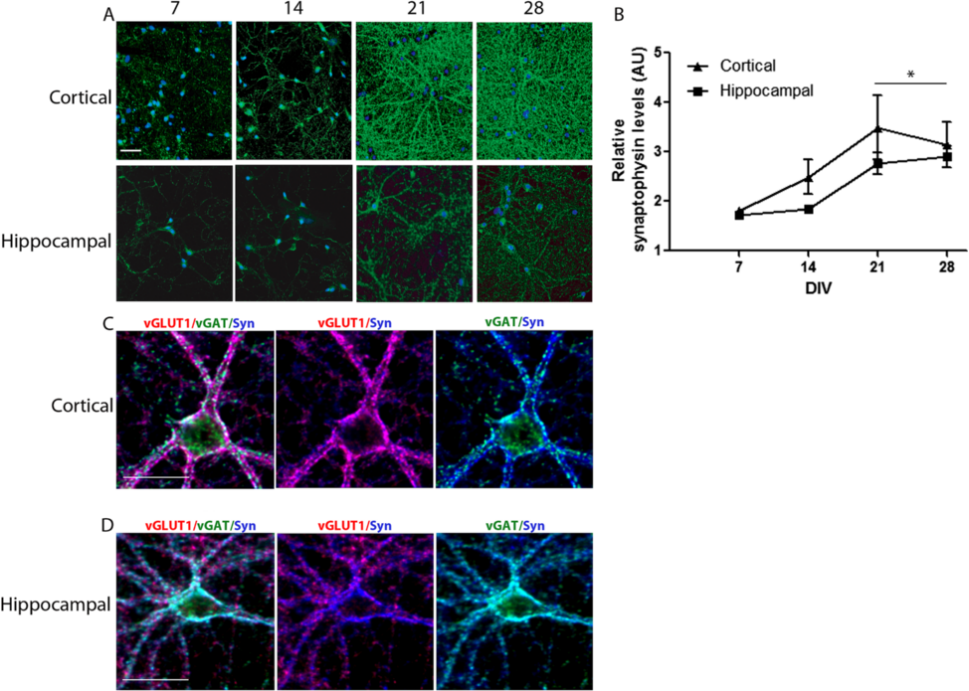
**Ontogeny of synaptophysin expression level in hippocampal and cortical cell cultures.** The level of synaptophysin expression increases with time in primary cultures of rat cortical and hippocampal neurons. Dissociated cell cultures were derived from the hippocampi and neocortices of rat pups on postnatal day 1 (P1). Cultures were either fixed and immunostained for synaptic proteins **(A, C, D)**, or cell lysates were collected for synaptophysin ELISA **(B)** at days *in vitro* (DIV) 7, 14, 21 and 28. **(A)** Representative fluorescence photomicrographs of synaptophysinsyn immunoreactivity at differing DIV in cortical or hippocampal neurons. Bar = 50 μm. **(B)** Relative ELISA levels confirm the increase in synaptophysin levels with increasing time in culture in both hippocampal and cortical neurons. AU = Arbitrary Units. Experiments were repeated >3 times with independent biological replicates. Data presented as the mean ± SD. Two-way ANOVA demonstrated a significant main effect of time and no main effect of cell type, therefore data from both cell types were collapsed within each time point for *post-hoc* analysis. *Significantly different from measurements at 7 DIV (Sidak’s *post-hoc* test, *p* < 0.05). **(C, D)** Representative fluorescence photomicrographs of a cortical **(C)** and hippocampal **(D)** neuron triple-labeled for synaptophysin, vGLUT1 and vGAT immunoreactivity at 21 DIV. Bar = 30 μm.

Synaptophysin has previously been used as a biomarker of synaptogenesis in cultured hippocampal neurons [14]. Total synaptophysin levels were utilized as a general marker of synaptogenesis and assessed qualitatively by immunocytochemistry (ICC) and quantitatively by ELISA over 28 days in culture. Synaptophysin immunoreactivity increased steadily with increasing days *in vitro* (DIV) in both hippocampal and cortical cultures (Figure 1A). Synaptophysin immunoreactivity was apparent in cell bodies and along processes, with the latter appearing punctate. The network of synaptophysin immunoreactive processes appears denser and more complex in cortical cell cultures relative to hippocampal cell cultures at any given time point (Figure 1A).

Data generated using a synaptophysin-specific ELISA normalized to total protein, similarly demonstrates increasing levels of synaptophysin with increasing time *in vitro* in both hippocampal and cortical cultures (Figure 1B). Synaptophysin ELISA data were analyzed by two-way analysis of variance (ANOVA) using cell type and time as the independent factors. A significant main effect of time (F_3,19_ = 7.823, *p* = 0.0013) was observed with no main effect of cell type or interaction. *Post hoc* analysis demonstrated that synaptophysin levels at 21 and 28 DIV were significantly increased relative to earlier time points.

To qualitatively assess whether the synaptophysin immunoreactivity recognized both excitatory and inhibitory synapses, we co-labeled rat hippocampal and cortical cultures for synaptophysin, vGLUT1 and vGAT, which are expressed in the presynaptic terminals of excitatory and inhibitory synapses, respectively [4-6]. As demonstrated in DIV 21 cultures (Figure 1C), synaptophysin immunoreactive puncta (blue) are predominantly co-localized with either vGLUT1 (red) or vGAT (green) immunoreactive puncta along dendrites. Interestingly, synaptophysin does not co-localize with vGAT immunoreactive puncta localized to the neuronal cell body. Similar patterns were observed in cultures immunostained at DIV 7, 14 and 28 (data not shown).

### High content imaging (HCI) of excitatory and inhibitory synapses

**Figure 2:**
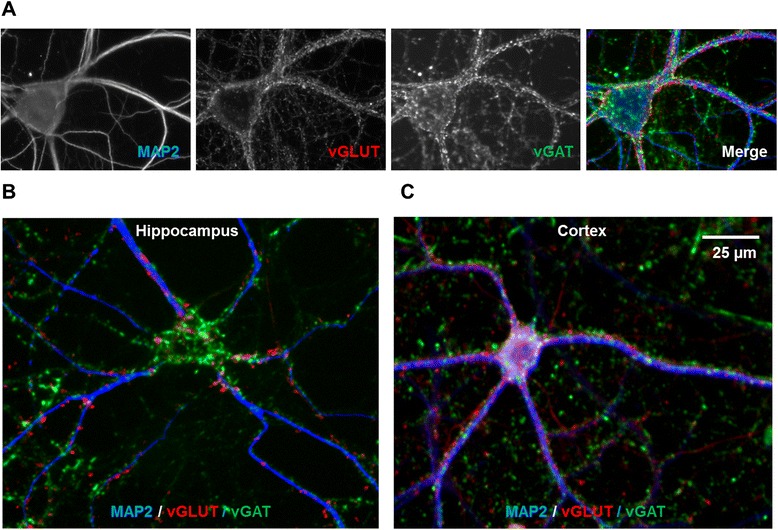
**Excitatory and inhibitory synapse localization in hippocampal and cortical neurons. (A)** Matching images of MAP2, vGLUT1 and vGAT immunolabeling in a hippocampal neuron. The panel to the far right is a pseudo-colored composite image of the three labels. **(B)** Pseudo-colored image of excitatory and inhibitory synapses contacting a MAP2 immunopositive hippocampal neuron at DIV 21. Inhibitory synapses (vGAT, green) preferentially contact the neuronal cell body. **(C)** Pseudo-colored image of excitatory and inhibitory synapses contacting a MAP2 immunopositive cortical neuron at DIV 21. Compared to hippocampal neurons, excitatory and inhibitory synapses are more evenly distributed along the dendrites of cortical neurons. Scale bar = 50 μm.

To quantify excitatory *versus* inhibitory synapses, we used HCI analysis of hippocampal and cortical cultures immunostained for the synaptic vesicle-associated proteins vGLUT1 and vGAT. Hippocampal and cortical neurons at 21 DIV were immunolabeled with antibodies specific for microtubule-associated protein 2 (MAP2) to identify dendrites (blue), vGLUT1 to mark excitatory synapses (red) and vGAT to distinguish inhibitory synapses (green) (Figure 2). In hippocampal neurons, vGAT immunopositive puncta are found primarily in contact with the cell body (Figure 2B). In contrast, vGLUT1 immunopositive puncta are localized primarily along the dendrites. In cortical neurons, the subcellular distribution of the two synaptic sub-types is less distinct. Both inhibitory and excitatory synapses are more evenly distributed across the cell body and dendrites.

**Figure 3:**
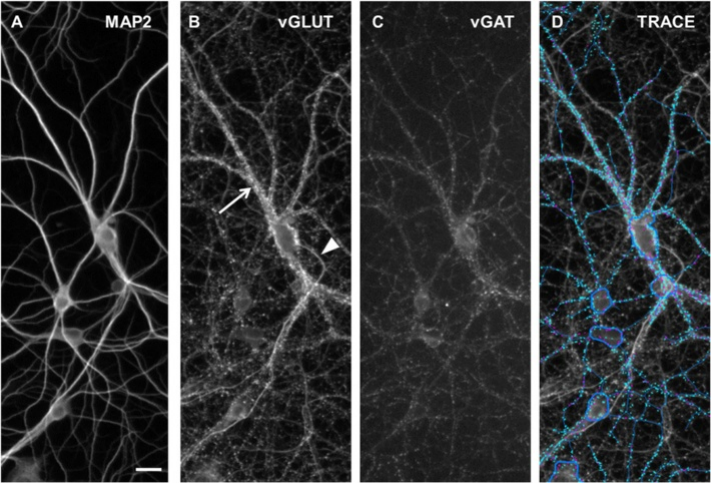
**Illustration of synaptogenesis high content imaging algorithm.** All panels are matching images from the same field-of-view in a hippocampal culture at DIV 21. **(A)** MAP2 immunoreactivity is localized to the dendrites and cell body (note: axons are not immunoreactive for MAP2). **(B)** Cultures immunostained for vGLUT1 exhibit two patterns of vGLUT1 immunolabeling: punctuate labeling along dendrites (arrow) and continuous low intensity labeling along MAP2 immunonegative axons (arrowhead). **(C)** Inhibitory synapse labeling with vGAT. **(D)** MAP2 immunopositive cell bodies are masked (dark blue shapes) and MAP2 immunopositive dendrites emanating from selected cell bodies are traced (blue lines). Excitatory (teal) and inhibitory (purple) synaptic puncta are only quantified if they contact a dendrite or are contained within the cell body mask. Scale bar = 25 μm.

HCI analysis has been used previously to quantify the number of synapses contacting the neuronal cell body and dendrites in cultured neurons as identified using synapsin immunoreactivity [10]. In this previous study of 12 DIV cortical neurons, we observed that punctate synapsin I immunoreactivity in close proximity to MAP2 immunopositive neurites (i.e., dendrites) was also in close proximity to PSD95 [10]. In contrast, synapsin I immunoreactivity not associated with dendrites did not co-localize with PSD95. In the present work, this HCI analysis method was adapted to simultaneously measure excitatory and inhibitory synapses in hippocampal and cortical neurons co-labeled for MAP2, vGLUT1 and vGAT using fluorophores with distinct excitation/emission spectra, as illustrated in Figure 3. Analysis of cultures immunolabeled with binary combinations of MAP2 + vGLUT1 and MAP2 + vGAT yielded results comparable to those of age-matched cultures with triple labeling for MAP2, vGLUT1 and vGAT (data not shown).

**Figure 4:**
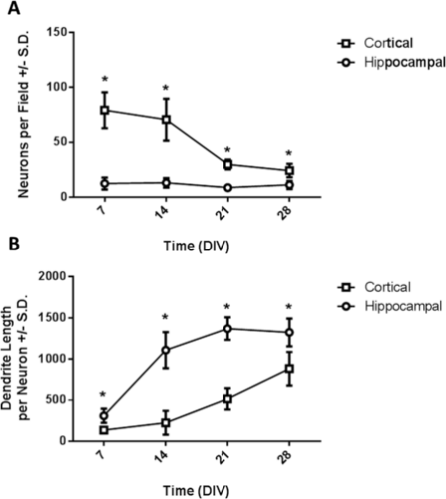
**Dendritogenesis in primary hippocampal and cortical neurons.** The number of neurons per field **(A)** and dendrite length **(B)** were measured in hippocampal (circles) and cortical (squares) neurons at 7, 14, 21 and 28 DIV. Data were analyzed by two-way ANOVA followed by *post hoc* tests to compare means at each time point within each cell type and means for each cell type within each time point. Time points labeled with an asterisk indicate that means differed between hippocampal and cortical neurons at that time point (Sidak’s multiple comparison test, *p* <0.05). All data are expressed as mean ± SD (n = 6–18 wells collected across 3–4 replicate cultures).

The average number of neurons per field was quantified for both hippocampal and cortical cultures as a function of time *in vitro* (Figure 4A). Note that cortical neurons were plated at a higher initial seeding density than hippocampal neurons. The number of hippocampal neurons per field did not change between 7 and 28 DIV. In contrast, the number cortical neurons per field decreased significantly between 14 and 21 DIV. The data were analyzed by two-way ANOVA with time and cell type as the independent factors. A significant interaction of time and cell type was observed (F_3,81_ = 35.26, *p* < 0.0001). *Post-hoc* analysis demonstrated that the number of cortical neurons per field was greater than the number of hippocampal neurons per field at each time point (Figure 4A, asterisks).

HCI analysis of MAP2 immunopositive processes was used to quantify dendrite length as a function of time in culture in each cell type (Figure 4B). In both hippocampal and cortical neurons, total dendrite length per neuron increased between 7 and 28 DIV. The amount of dendrite outgrowth was more pronounced in hippocampal neurons relative to cortical neurons. Dendrite outgrowth data was analyzed by two-way ANOVA with time and cell type as the independent factors. A significant interaction of time and cell type was observed (F_3,81_ = 30.99, *p* < 0.0001) indicating that the time course of dendrite outgrowth was different between the two cell types. *Post-hoc* analysis demonstrated that total dendrite length was greater in hippocampal neurons relative to cortical neurons at each time point (Figure 4B, asterisks). In hippocampal neurons, dendrite length increased significantly beginning at 14 DIV and then plateaued between 21 and 28 DIV (Figure 4B, circles). In cortical neurons, significant increases in dendrite length were not observed until 21 DIV and continued to increase through 28 DIV, the final time point examined (Figure 4B, squares).

**Figure 5:**
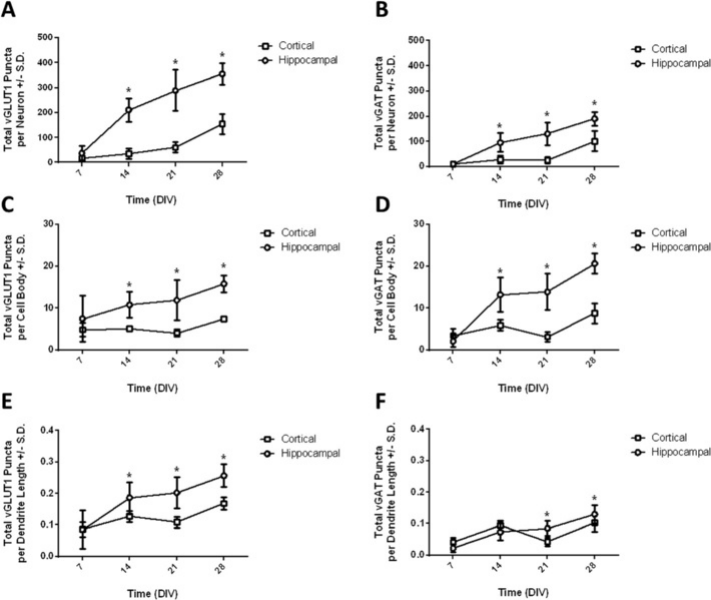
**Excitatory and inhibitory synapse development in hippocampal and cortical neurons as assessed using high content image analysis.** Quantification of excitatory (left column) and inhibitory (right column) synaptogenesis in hippocampal (circles) and cortical (squares) using high content image analysis. The number of vGLUT1 or vGAT immunopositive puncta was used to measure the number of excitatory and inhibitory synapses, respectively. **(A)** Total number of vGLUT1 puncta per neuron. **(B)** Total number of vGAT puncta per neuron. **(C)** Total number of vGLUT1 puncta per cell body. **(D)** Total number of vGAT puncta per cell body. **(E)** Total number of vGLUT1 puncta per μm dendrite length. **(F)** Total number of vGAT puncta per μm dendrite length. Data were analyzed by two-way ANOVA. For each endpoint, a significant interaction between time and cell type was observed; therefore, *post hoc* mean contrast tests were performed to compare means within each cell type across time and means between cell types within each time point. *Means are significantly different between cell types within a time point (Sidak’s test, *p* < 0.05). All data are expressed as mean ± SD (n = 6–18 wells collected across 3–4 replicate cultures).

Quantitative data of excitatory and inhibitory synapse numbers in hippocampal and cortical neurons (Figure 5) were analyzed by two-way ANOVA with time and cell types as the independent factors. A significant interaction between time and cell type (F_3,81_ = 37.07, *p* < 0.0001) was observed for the total number of excitatory synapses per neuron (i.e., vGLUT1 immunopositive puncta) (Figure 5A). The total number of excitatory synapses increased over time in both hippocampal and cortical neurons, although differences in the ontogeny of synaptogenesis between the respective cell types were apparent. Total excitatory synapses increased significantly between 7 and 14 DIV in hippocampal neurons and continued to increase to 28 DIV. In contrast, in cortical neurons, significant increases in total excitatory synapse number were not observed until 21 DIV and continued to increase up to 28 DIV.

A significant interaction between time and cell type (F_3,81_ = 17.86, *p* < 0.0001) was also observed for the total number inhibitory synapses per neuron (i.e., vGAT puncta) (Figure 5B). Similar to excitatory synapses, the total number of inhibitory synapses increased over time in both cell types, but the temporal profile of inhibitory synaptogenesis varied between cell types. Total inhibitory synapses increased significantly between 7 and 14 DIV in hippocampal neurons and continued to increase steadily out to 28 DIV. In cortical neurons, significant increases in total inhibitory synapse numbers were not observed until 28 DIV.

A significant interaction between time and cell type (F_3,81_ = 3.67, *p* = 0.0155) was observed for the number of excitatory synapses per cell body (Figure 5C). In hippocampal neurons, the number of excitatory synapses per cell body increased steadily between 7 and 28 DIV, although the magnitude of the increase was small (<2-fold). No significant increase in the number of excitatory synapses per cell body was observed in cortical neurons.

Similarly, a significant interaction between time and cell type (F_3,81_ = 35.05, *p* < 0.0001) was observed for the number of inhibitory synapses per cell body (Figure 5D). In comparison to excitatory synapses, increases in the number of inhibitory synapses per cell body were of greater magnitude. In hippocampal neurons, the number of inhibitory synapses per cell body increased steadily over time beginning at 14 DIV and increased by ~10-fold by 28 DIV. In cortical neurons, significant increases in the number of inhibitory synapses per cell body were not observed until 28 DIV. The marked increase in inhibitory synapse number in the cell body of hippocampal neurons but not cortical neurons is consistent with the qualitative observations presented in Figure 2.

A significant interaction between time and cell type (F_3,81_ = 6.83, *p* = 0.0004) was observed for the number of excitatory synapses per dendrite length (Figure 5E). In hippocampal neurons, the number of excitatory synapses per dendrite length increased steadily over time out to 28 DIV. The number of excitatory synapses per dendrite length also increased over time in cortical neurons, with a slight but significant increase at 14 DIV, followed by an additional increase at 28 DIV.

A significant interaction between time and cell type (F_3,81_ = 11.78, *p* < 0.0001) was also observed for the number of inhibitory synapses per dendrite length (Figure 5F). In both cell types, the increases in inhibitory synapses per dendrite length were less pronounced than the increases in excitatory synapses per dendrite length. In hippocampal neurons, the number of inhibitory synapses per dendrite length began to increase at 14 DIV and continued to increase throughout 28 DIV. In cortical neurons, a significant increase in the number of inhibitory synapses per dendrite length was noted at 14 DIV with additional increases at 28 DIV. In both cell types, the number of inhibitory synapses per dendrite length was less than the number of excitatory synapses per dendrite length.

**Figure 6:**
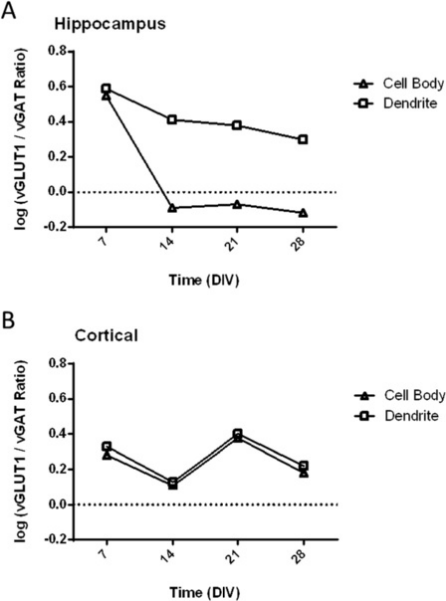
**Ratio of excitatory to inhibitory synapses in the cell body** ***versus*** **dendritic compartments of hippocampal and cortical neurons.** The ratio of vGLUT1 to vGAT immunopositive synaptic puncta in the cell body and dendritic compartments of hippocampal **(A)** and cortical **(B)** neurons is expressed as the log10 (# of vGLUT1 puncta/# of vGAT puncta). The number of inhibitory synapses is higher than that of excitatory synapses in the cell body compartment of hippocampal neurons. In contrast, the number of excitatory synapses is higher than that of inhibitory synapses in the dendritic compartment of hippocampal neurons. In both the dendritic and cell body compartments of cortical neurons, the number inhibitory and excitatory synapses were similar.

The ratio of excitatory to inhibitory synapses in the cell body compartment and the dendrite compartment was also analyzed (Figure 6). Data in Figure 6 are expressed as log_10_ (# of vGLUT1 puncta/# of vGAT puncta). Values above 0 indicate that more excitatory than inhibitory synapses whereas values below 0 indicate the opposite, e.g., inhibitory synapses are more numerous than excitatory synapses. In hippocampal neurons, vGLUT1 immunopositive synapses were more numerous than vGAT immunopositive synapses in the dendrite compartment at all times examined. In contrast, vGAT synapses became more numerous than vGLUT1 synapses in the cell body compartment at time points beyond 7 DIV (Figure 6A). In cortical neurons, vGLUT1 synapses were more numerous than vGAT synapses in both the dendrite and cell body compartment at all times examined (Figure 6B). These data indicate that there may be differences in the amount of inhibitory synaptic tone between hippocampal and cortical neurons.

### Microelectrode array (MEA) analysis

**Figure 7:**
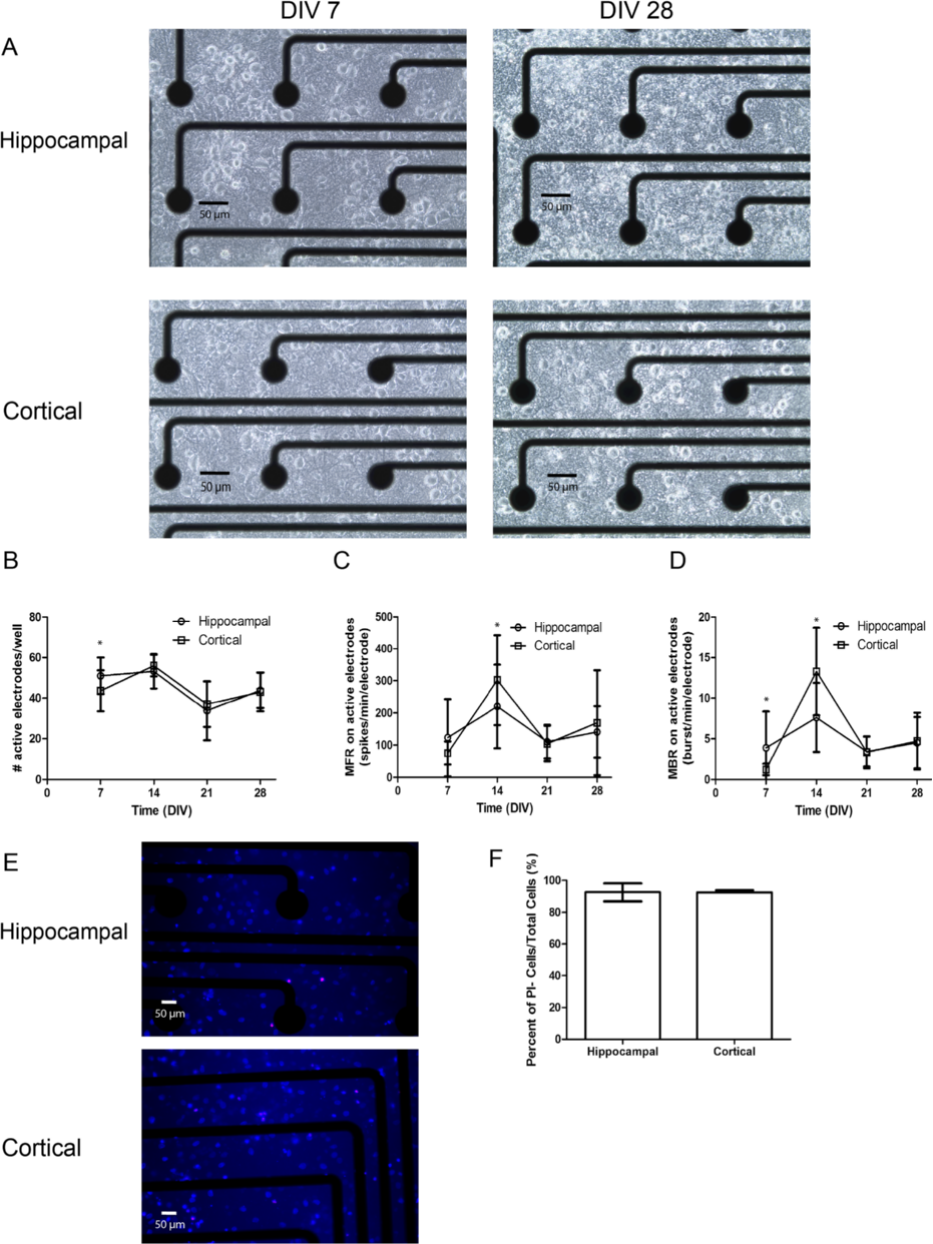
**Developmental profile of network activity in hippocampal and cortical cultures. (A)** Representative phase-contrast images of cells dissociated from P1 rat hippocampi or cortices and grown on microelectrode arrays (MEAs) at a density of 150,000 cells/MEA. Recordings of spontaneous electrical activity were collected every 7 days beginning on DIV 7 and continuing through DIV 28. Active electrodes were defined as electrodes with an average of ≥; 5 spikes/min; inactive electrodes with < 5 spikes/min were excluded from analysis. Burst analysis was performed using Neuroexplorer (Version 3.2, NEX Technologies, Littleton, MA, USA), with a burst defined as a minimum of 4 spikes lasting 0.02 s with 0.1 s between bursts. Network activity as a function of DIV was measured as **(B)** number of active electrodes/well; **(C)** mean firing rate (MFR); and **(D)** mean bursting rate (MBR). Data were analyzed by two-way ANOVA. For each endpoint, a significant interaction between time and cell type was observed; therefore, *post hoc* mean contrast tests were performed to compare means within each cell type across time and means between cell types within each time point. *Means are significantly different between cell types within a time point (Sidak’s test, p < 0.05). All data are expressed as mean ± SD (12–24 wells across four 12-well MEA plates from two independent dissections). **(E)** Representative images of single wells within MEAs stained with Hoechst 33342 (blue) and propidium iodide (PI, pink) at the end of the recording period on 28 DIV. **(F)** Three random sites were imaged within each well to quantify the percentage of viable cells, which was determined using the following: [(# Hoechst-stained cells) – (# PI stained cells)]/(# Hoechst-stained cells) × 100. The difference in percent cell viability between 28 DIV hippocampal and cortical cultures were not significantly different.

Representative images of hippocampal and cortical cultures plated on MEAs demonstrate a qualitative increase in the complexity of the cultures between 7 and 30 DIV (Figure 7A). The total number of active electrodes (Figure 7B), mean firing rate (Figure 7C) and mean burst rate (Figure 7D) were measured between 7 and 28 DIV. Data were analyzed using two-way ANOVA with time and cell type as the independent factors. A significant interaction between time and cell type (F_3,180_ = 2.93, *p* = 0.0349) was observed for the number of active channels (Figure 7B). At DIV 7, the number of active channels per well was greater in hippocampal neurons than in cortical neurons. In both cell types, the number of active channels decreased between 14 and 21 DIV.

A significant interaction between time and cell type (F_3,180_ = 3.188, *p* = 0.0250) was observed for mean firing rate (Figure 7C). At DIV 14, the mean firing rate was higher in cortical neurons than in hippocampal neurons. In both cell types, the mean firing rate increased between 7 and 14 DIV followed by a decrease at 21 DIV. There was no significant difference between 21 and 28 DIV. A significant interaction between time and cell type (F_3,180_ = 12.04, *p* < 0.0001) was also observed for mean burst rate (Figure 7D). In both hippocampal and cortical neurons, the mean burst rate increased between 7 and 14 DIV and then at later time points decreased to levels below those observed at 14 DIV. Cell viability was determined in both culture models following the last MEA recording at 28 DIV. As determined using Hoechst staining to identify all cells and staining with propidium iodide (PI) to identify dead cells (Figure 7E), there were no significant differences in cell viability between hippocampal and cortical neurons (Figure 7F).

## Discussion

Primary cultures of rodent neurons from various brain regions, including the cortex and hippocampus, have been powerful tools for elucidating the cellular and molecular mechanisms that control synapse formation and stabilization. However, the reported rates of synaptogenesis *in vitro* vary considerably between studies depending upon the biomarkers, experimental models and experimental methods used to identify and quantify synapses [15]. In the present study, we compared the ontogeny of synapse development in primary rat cortical and hippocampal cell cultures using three complementary methods: 1) quantification of the levels of synaptophysin protein by ELISA; 2) quantification of excitatory and inhibitory synapse number using HCI; and 3) quantification of synaptic network activity using MEAs. Each method demonstrated a general increase in synapses over time in both culture models. However, assessment at the cellular level using HCI demonstrated distinct differences between neuronal cell types with respect to the temporal profile of synapse development and the subcellular distribution of excitatory and inhibitory synapses. The functional significance of these differences was confirmed by the assessment of network activity using MEAs.

One of the challenges encountered in this study was the need to use different cell plating densities between cell types and experimental platforms. The basal rate of neuronal cell loss was greater in cortical cultures than hippocampal cultures, necessitating the use of higher cortical cell densities relative to hippocampal cell densities for the synaptophysin ELISA and high content imaging studies. Loss of cells over time has been observed previously in neuronal cultures [16], although to our knowledge, differences in basal neuronal attrition rates in culture have not been systematically examined. In addition, more densely plated cultures were required to produce reliable measurements of synapse connectivity on the MEA platform as compared to cultures used for either ELISA or HCI experiments. These observations highlight the need for cell type and platform specific optimization of seeding densities to ensure to collection of high quality data.

The need to use different plating densities between cell types and across platforms complicates comparisons of the temporal profile of synaptogenesis. However, it must be noted that within each cell type, the same plating density was used for cultures prepared for ELISA and HCI experiments, thereby allowing direct comparisons of data obtained for each cell type across these two platforms. While it may be difficult to compare the timing of synapse formation between cell types, the general spatiotemporal patterns of excitatory and inhibitory synapse development are likely unaffected by differences in plating density. Moreover, hippocampal and cortical cultures were plated at equivalent densities for the MEA experiments, enabling comparisons between cell types with respect to ontogenetic profiles of synaptic activity. Even in light of these potential confounders, parallel use of complimentary methods for quantifying synaptogenesis will likely yield a more comprehensive understanding of responses to experimental manipulations or stressors (i.e., chemical exposure).

Synaptophysin is an integral membrane protein of synaptic vesicles whose expression at both the transcript and protein level increases as neurons mature [14,17]. Thus, synaptophysin has been used as a biomarker of presynaptic terminals *in vivo*, and as a biomarker of synaptogenesis in cultured hippocampal neurons [18,19]. Using both synaptophysin immunocytochemistry and ELISA as a general marker for synaptogenesis, we observed that the amount of synaptophysin increased in both hippocampal and cortical cultures with increasing time in culture. The fold-increase in synaptogenesis we observed using this approach is similar to that reported in other studies that used ELISA or Western blotting to quantify synaptophysin levels in neurons cultured under similar conditions [14,20]. Similarly, we had previously reported increased expression levels of synapsin, another synaptic vesicle protein, in cultured cortical neurons with increasing time in culture [10]. Qualitative comparisons of synaptophysin immunoreactivity between culture types suggested that cortical cultures express significantly more synaptophysin than hippocampal neurons at any given time in culture. This is consistent with a prior report that synaptophysin levels are higher in cortical *versus* hippocampal neurons cultured from embryonic mice [21]. However, analysis of synaptophysin expression level by ELISA (in which equal amounts of protein were loaded per sample) revealed no cell type-specific differences in synaptophysin expression level at any given time point. These data demonstrate that the amount of synaptophysin protein per amount of total protein did not differ between the culture models and suggests that the amount of synaptophysin protein produced on a per cell basis is not different between hippocampal and cortical neurons in culture.

Our findings are consistent with the literature that the expression of synaptophysin correlates well with neuronal maturation; however, because not all synaptic vesicles are localized to synapses, the immunocytochemical localization of synaptophysin does not necessarily indicate a true synapse [22]. Furthermore, quantification of synaptophysin expression level by either immunocytochemistry or ELISA provides no information regarding the type of synapses formed or their function [23], as we confirmed in hippocampal and cortical cultures immunostained for synaptophysin, vGLUT1 and vGAT. To address this issue, we applied high-throughput, HCI technology to quantify vGLUT1 and vGAT immunopositive puncta. To increase the likelihood that the vGLUT1 and vGAT immunopositive puncta included in our analysis represent the pre-synaptic half of a bipartite synapse, we quantified puncta that were immunopositive for these presynaptic proteins that co-localized with MAP2, a biomarker of postsynaptic structures, specifically dendrites and neuronal cell bodies.

The development of the dendritic arbor is intimately tied to synapse formation. Synaptic connections increase in parallel to dendritic development, and abnormalities in dendritic length are associated with changes in synapse number and function [24-29]. In our studies, dendrite length increased with increasing time in culture. On a per cell basis, the amount of dendritic growth was greater in hippocampal than in cortical neurons at each time point. This may reflect the fact that cortical cells were plated at a higher initial seeding density than the hippocampal cells, thus reducing the distance required for cortical dendrites to grow before contacting a neighboring neuron. Alternatively, inherent differences in the rate of *in vitro* dendritic growth between the two cell types may contribute to this effect.

The numbers of vGLUT1 and vGAT immunopositive puncta also increased in hippocampal and cortical cell cultures with increasing time in culture, which is consistent with previous experiments performed in our lab [10] and with reports in the literature [30,31]. However, the ontogenetic profile of these parameters differed depending on neuronal cell type in that significant increases in the numbers of vGLUT1 and vGAT immunopositive puncta increased much earlier in hippocampal neurons relative to cortical neurons. This is consistent with *in vivo* observations that rates of synaptogenesis differ between and within brain regions even at similar stages of brain development [32,33]. Another possible explanation for the differences observed in our culture models is that cortical cell cultures have a broader distribution of neuronal types; whereas, in hippocampal cell cultures, pyramidal neurons are the predominant neuronal cell type [34-36]. This could likewise explain the more prominent numbers of vGLUT1 puncta observed in the hippocampal neurons as vGLUT1 has been shown to localize preferentially in the stratum pyramidale [37]. However, the effect of cell density on the time course of synaptogenesis between the two culture types cannot be disregarded. While cell density does not appear to be a contributor in the determination of the composition of neuron cell types *in vitro*, lower cell densities result in faster development of synapses and higher synapse-to-neuron ratios [38]. This could explain the slower onset of synaptogenesis in the cortical cultures despite their higher cell density. Furthermore, the influence of our culture reagents and conditions must be taken into account, as they can have a robust and differential effect on neurite outgrowth in different neuronal cell populations [39].

In addition to cell type-specific differences in the ontogeny of synaptogenesis, there was a significant difference in the number and ratio of excitatory and inhibitory synapses in hippocampal *versus* cortical cell cultures. In general, there were significantly more excitatory and inhibitory synapses formed in hippocampal cell cultures relative to cortical cell cultures beginning on DIV 14 through DIV 28. With regards to the ratio of excitatory to inhibitory synapses, previous studies of hippocampal neurons have reported that the number of excitatory synapses generally is greater than the number of inhibitory synapses in mature neuronal cell populations [40,41]. Our data are consistent with these prior studies with respect to the total number of excitatory *versus* inhibitory synapses and the ratio of excitatory to inhibitory synapses that form on MAP2 immunopositive dendrites. The one notable exception to this generalization was the ratio of excitatory to inhibitory synapses formed on neuronal cell bodies in hippocampal cell cultures. The number of vGAT immunopositive puncta formed on neuronal cell bodies was significantly higher in hippocampal *versus* cortical neurons, and the ratio between these two synaptic types was such that vGAT immunopositive puncta outnumbered vGLUT1 immunopositive puncta at DIV 14 through 28. Inhibitory synapse input predominates in pyramidal cell somata and it has been speculated this is to match the inhibitory efficacy in dendrites, due to the relatively larger diameter of the pyramidal cell somata [14,15]. Thus, the present findings are consistent with previous literature and can perhaps again be attributed to the differing cell compositions of the cultures [42].

The relative numbers of excitatory and inhibitory synapses are a critical determinant of network activity, which is the functional readout of synaptogenesis. To determine whether the data we obtained from high content imaging of excitatory *versus* inhibitory synapses is predictive of network activity, we also measured synaptic function by recording activity in hippocampal and cortical cultures plated onto MEAs. MEAs enable simultaneous, noninvasive extracellular recording over long periods of time in a relatively high-throughput format compared to traditional electrophysiological techniques. Cultured neurons can be followed from the time of isolation until the development of spontaneous firing, providing a unique opportunity to record the ontogeny of neuronal network activity. These measurements have a wide variety of applications, from basic research to drug discovery and toxicology screening [13,43,44]. While there are numerous studies investigating neuronal networks of specific neuronal cell populations [45,46], studies comparing the development of neuronal network activity between different neuronal cell types are lacking. In our experiments, the mean firing rate and mean bursting rate, both classical descriptors of macroscopic network activity state, were followed over time [47,48]. In both culture models, network activity was evident as measurable spike and burst activity by DIV 7, and this significantly increased by DIV 14, followed by a decrease at subsequent time points. This pattern of an increase in firing activity followed by a transient reduction is consistent with previous literature, although these shifts occurred earlier in our studies [9,49]. A possible explanation for the earlier peak and fall of firing activity is the comparatively high density of neurons utilized in our study (~235,000 cells/cm^2^). Culture density has consistently been shown to have significant influence on the development, localization, and function of synapses, with higher density cultures exhibiting earlier developmental onset of network activity [38,50,51]. Thus the ontogeny of network activity in our studies is comparable to studies that utilize similar higher-density cultures [52] .

Interestingly, the ontogeny of network activity was similar between the two culture models; however, at DIV 14, cortical cultures displayed significantly higher firing rates and burst activity than hippocampal neurons. This difference may reflect the higher vGLUT1/vGAT ratio observed in the cell body compartment of cortical neurons relative to hippocampal neurons. Inhibitory GABAergic input modulates firing behavior such that decreased GABAergic neurotransmission shifts firing to a bursting pattern [53,54], and a lower ratio of excitatory to inhibitory input can depress bursting behavior in cultured neurons [55]. The time at which the cell type-dependent differences in bursting behavior manifest may reflect differences in the rate of maturation of *in vitro* excitatory connections [56,57]. However, a differential distribution of neuronal cell types or glial cells between the two culture models cannot be discounted as contributing to the differences in bursting behavior we observed in hippocampal *versus* cortical cultures, as all of these factors can influence neuronal network activity [58].

## Conclusions

In summary, these data provide a comparison of the ontogeny of *in vitro* synaptogenesis between hippocampal and cortical neuronal cell cultures. Hippocampal neurons were observed to mature faster than cortical cell cultures, as evidenced by increased dendritic lengths and numbers of vGLUT1 and vGAT immunopositive puncta at any given DIV. Another marked difference between the two culture models was the decreased excitatory to inhibitory synaptic input to the cell soma in hippocampal neurons *versus* cortical neurons. Cortical neurons in general exhibited a slower developmental timeline, showing more robust increases at later time points. Indeed, it appeared that dendritic length and vGLUT1/vGAT expression had not yet plateaued in cortical cell cultures by the end of the experimental period. At the level of network activity, hippocampal and cortical cells displayed remarkable similarity in the ontogeny of firing rates; however, cortical neurons exhibited significantly higher burst activity at DIV 14, which correlated with spatiotemporal differences in the relative ratio of excitatory to inhibitory synapses between the two culture models. Collectively, our data indicate that the ontogeny of *in vitro* synaptogenesis observed in the present study is largely consistent with data reported in previous studies and that cell type-dependent differences exist in synaptogenic profiles.

These results further suggest that utilization of multiple techniques can provide a more integrative view of synaptogenesis, which may provide more useful and predictive insight of how chemical exposures and pharmacologic interventions influence this critical neurodevelopmental endpoint. Synaptophysin ELISA may be useful for assessing maturation of cultures but is not very useful for providing detailed information about synapse types or functionality. HCI can provide quantitative information regarding the types of synapses being formed and their subcellular distribution but does not provide information regarding synaptic function. MEAs provide measurements of synaptic activity, but changes in underlying cellular events that influence activity measurements (i.e., synaptic number, localization or even loss of neurons due to cell death) cannot be determined. Our findings demonstrate that high-throughput immunocytochemical assays to measure excitatory and inhibitory synapse formation, in parallel with functional studies of neuronal activity using MEA recordings, can be used to study the effect of environmental chemicals and/or drugs on synaptic network formation and function.

## Methods

### Animals

Animals were treated humanely and with regard for alleviation of suffering according to protocols approved by the Institutional Animal Care and Use Committee of the University of California, Davis. Timed pregnant Sprague Dawley rats were purchased from Charles River Laboratory (Hollister, CA) and individually housed in standard plastic shoe box cages with corn cob bedding in a temperature (22 ± 2°C) controlled room on a 12 h reverse light-dark cycle. Food and water were provided *ad libitum*.

### Cell culture

Primary cultures of dissociated cortical and hippocampal cells were prepared and maintained as previously described [59]. Briefly, the neocortex and hippocampus of postnatal day 0–1 rat pups were dissociated and plated onto tissue culture plates precoated with poly-L-lysine (molecular weight 300,000, Sigma, St. Louis, MO). For ICC studies, cell suspensions were plated onto CoStar® 96-well plates (Corning, Inc, Corning, NY); for ELISA studies, on Nunc® 6-well polystyrene plates (Thermo Scientific, Waltham, MA). For the ICC and ELISA studies, cortical cells were plated at a density of 78,000 cells/cm^2^ while hippocampal neurons were plated at a density of 31,250 cells/cm^2^. For analysis of network activity, dissociated cells were plated on 12-well polystyrene MEA plates in which each well contained 64 nanoporous platinum electrodes (Axion Biosystems, Inc., Atlanta, GA). MEAs were precoated with poly-L-lysine (0.5 mg/ml, Sigma) and laminin (10 μg/ml, Invitrogen, Carlsbad, CA) and cells were plated at a density of 235,785 cells/cm^2^. All cultures were maintained in Neurobasal-A (Invitrogen) supplemented with 2% B27 (Invitrogen) and 2 mM Glutamax (Invitrogen). At DIV 4, cytosine-D-arabinofuranoside (Sigma) was added to the medium at a final concentration of 5 μM. Half of the medium was replaced with fresh Neurobasal-A supplemented with B27 once weekly.

### Synaptophysin ELISA

The relative levels of synaptophysin were quantified in primary cultures of cortical and hippocampal cells plated on 6-well plates and maintained as described above. Cell lysates were collected at 7, 14, 21 and 28 DIV in 150 μL of RIPA buffer (50 mM Trizma Base, 150 mM NaCl, 2 mM EDTA, 1% Triton X-100, and 0.1% SDS, pH 7.4) containing HALT protease and phosphatase inhibitors (Thermo Scientific, Rockford, IL). Samples were centrifuged and then stored at -80°C. Sandwich ELISAs using antibodies specific for synaptophysin were adapted from a previously described method [60]. Briefly, total protein was determined using Pierce BCA protein assay (Thermo Scientific) and 10 μg of each sample was loaded in plates with capture antibody that specifically recognizes synaptophysin (1:250, clone SY38, MAB368, Millipore, Temecula, CA). This amount of protein was determined in pilot studies with adult Sprague-Dawley whole brain homogenate in RIPA buffer diluted 1:20 then 1:2 to generate a linear response in the ELISA. Detection of 3,3′,5,5′-tetramethylbenzidine was measured at 450 nm absorbance on a spectrophotometer microplate reader (BioTek Instruments, Winooski, VT). Samples were run in triplicate using three independent biological replicates. Sample values were normalized to blank controls within each plate.

### Immunocytochemistry

Cortical and hippocampal cell cultures were fixed in 4% paraformaldehyde containing 4% sucrose for 1 h. Cultures were incubated with Hoechst Stain 33258 (3 μg/mL) (Invitrogen) diluted in phosphate buffered saline (PBS, pH 7.4) for 5 min then rinsed with PBS and permeabilized in 0.2% Triton X-100 in PBS for 5 min. Cultures were then blocked with 5% bovine serum albumin in PBS for 20 min and incubated with primary antibodies overnight at 4°C. Primary antibody solutions included guinea pig anti-MAP2 (1:800, Synaptic Systems, Göttingen, Germany), rabbit anti-vGLUT1 (1:1000, Synaptic Systems), mouse anti-vGAT (1:500, Synaptic Systems), rabbit anti-synapsin (1:500, Millipore, Billerica, MA) or mouse anti-synaptophysin (1:500, Dako, Carpinteria, CA). After extensive rinsing with PBS, cultures were incubated with Alexa-Fluor® secondary antibodies (1:500, Molecular Probe, Eugene, OR) for 2 h at room temperature. After washing with PBS to remove unbound secondary antibody, wells were filled with PBS at 4°C, sealed tightly with Parafilm® (Bemis, Neenah, WI) and shipped to the U.S.E.P.A. laboratories in Research Triangle Park, NC under refrigeration.

### High content image (HCI) analysis

Image acquisition and analysis were performed using a Cellomics® ArrayScan® V_TI_ (Thermo Scientific) as described previously [61]. Images were acquired using a Zeiss 20× objective (0.4 NA) and ORCA-ER CCD camera with 0.63× adaptor. Images were acquired in high resolution mode (1×1 pixel binning) with a resolution of 0.5 μm/pixel. For each well examined, twelve unique fields-of-view were sampled. Within each field, matched fluorescent images of Hoeschst-labeled nuclei, MAP2-Alexa Fluor® 647 immunolabeled neurons, vGLUT1-Alexa Fluor® 546 labeled excitatory puncta and vGAT-Alexa Fluor® 488 labeled inhibitory puncta were acquired using 365/515 (channel 1), 655/730 (channel 2), 549/600 (channel 3) and 475/515 (channel 4) nm excitation/emission filter couplings, respectively with an XF-93 dichroic mirror. In each channel, CCD camera exposure times for each channel were determined by surveying wells across multiple time points. Exposure times were held constant for all time points in the study.

Image analysis was performed using the Cellomics® Neural Profiling BioApplication. A previously developed high-content image analysis algorithm [10] was optimized using representative images of cortical and hippocampal neurons across multiple times points to identify inclusions/exclusion parameters for nucleus, cell body, dendrite and synaptic puncta identification, cell body masking, dendrite tracing and synaptic puncta. Manual comparison of representative images to matched tracing overlays was performed during optimization to insure the algorithm provided an accurate trace across all time points examined in this study. The step-wise image analysis scheme used in the present study is as described in Harrill et al. [10] with the modification that two distinct populations of synapses (i.e., excitatory, vGLUT1; inhibitory, vGAT) are quantified sequentially based upon contact with a MAP2 positive dendrite or cell body. A full listing of parameters used for the image analysis algorithm is available from the authors upon request.

The total number of vGLUT1 and vGAT puncta was categorized as either contacting the cell body (i.e., the postsynaptic contact site was located within a MAP2-immunopositive cell body mask) or contacting a dendrite (i.e., the postsynaptic contact site was located on a MAP2-immunopositive process). Endpoints analyzed in this study included: 1) the number of neurons per field; 2) dendrite length per neuron; 3) the number of vGLUT1 puncta per neuronal cell body; 4) the number of vGAT puncta per neuronal cell body; 5) the number of vGLUT1 puncta per μm dendrite length; 6) the number of vGAT puncta per μm dendrite length; 7) the total number of vGLUT1 puncta per neuron; and 8) the total number vGAT puncta per neuron. For each synaptic type, the total number of synaptic puncta includes both cell body- and dendrite-localized synapses. The ratio of excitatory to inhibitory (vGLUT1/vGAT) synapses was also calculated with respect to the number localized to cell bodies *versus* dendrites as well as total synapses (cell body + dendrites).

### MEA recording and analysis

Spontaneous network activity was recorded from the MEAs as previously described [62]. Data were recorded from Axion Biosystems’ Maestro 768-channel amplifier and Middle-man data acquisition interface using Axion’s Integrated Studio (AxIS 1.4.2). Recordings were collected for 33 min at 35°C under ambient atmospheric conditions, with the first 3 min of recording excluded from analysis. Recordings were passed through a Butterworth band-pass filter (300 Hz high pass cutoff, 5000 Hz low-pass cutoff) with a variable threshold spike detector of 6× standard deviation of each channel’s root mean square noise. Spike counts from active electrodes were quantified, with active electrodes defined as electrodes with activity of ≥5 spikes/min. Inactive electrodes were not included in any analysis. Burst analysis was performed using Neuroexplorer (Version 3.2, NEX Technologies, Littleton, MA, USA), with a burst defined as a minimum of 4 spikes lasting 0.02 s with 0.1 s between bursts.

### Statistics

Statistical analyses were performed using GraphPad Prism software (La Jolla, CA). Specific statistical tests are identified in the results section and in figure legends.

## Abbreviations

ANOVA: Analysis of variance
CNS: Central nervous system
DIV: Days *in vitro*
GABA: γ-aminobutyric acid
ELISA: Enzyme-linked immunosorbent assay
HCI: High-content imaging
MEA: Microelectrode array
PBS: Phosphate buffered saline
PSD95: Postsynaptic density 95
vGAT: Vesicular GABA transporter
vGLUT1: Vesicular glutamate transporter 1
